# Neuroprotective dobutamine treatment upregulates superoxide dismutase 3, anti-oxidant and survival genes and attenuates genes mediating inflammation

**DOI:** 10.1186/s12868-018-0415-2

**Published:** 2018-03-09

**Authors:** Tina Markus, David Ley, Stefan R. Hansson, Tadeusz Wieloch, Karsten Ruscher

**Affiliations:** 1 Department of Pediatrics, Lund University, Lund, Sweden; 2 Department of Obstetrics and Gynecology, Lund University, Lund, Sweden; 3 Laboratory for Experimental Brain Research, Division of Neurosurgery, Department of Clinical Sciences, Lund University, 22184 Lund, Sweden

**Keywords:** Dobutamine, Gene array, Hippocampal slice cultures, Hypoxia, Lipopolysaccharide, Preconditioning, Superoxide dismutase 3

## Abstract

**Background:**

Labor subjects the fetus to an hypoxic episode and concomitant adrenomodullary catecholamine surge that may provide protection against the hypoxic insult. The beta1-adrenergic agonist dobutamine protects against hypoxia/aglycemia induced neuronal damage. We aimed to identify the associated protective biological processes involved.

**Results:**

Hippocampal slices from 6 days old mice showed significant changes of gene expression comparing slices with or without dobutamine (50 mM) in the following two experimental paradigms: (1) control conditions versus lipopolysacharide (LPS) stimulation and (2) oxygen–glucose deprivation (OGD), versus combined LPS/OGD. Dobutamine depressed the inflammatory response by modifying the toll-like receptor-4 signalling pathways, including interferon regulatory factors and nuclear factor κ B activation in experimental paradigm 1. The anti-oxidant defense genes superoxide dismutase 3 showed an upregulation in the OGD paradigm while thioredoxin reductase was upregulated in LPS paradigm. The survival genes Bag-3, Tinf2, and TMBIM-1, were up-regulated in paradigm 1. Moreover, increased levels of SOD3 were verified on the protein level 24 h after OGD and control stimulation in cultures with or without preconditioning with LPS and dobutamine, respectively.

**Conclusions:**

Neuroprotective treatment with dobutamine depresses expression of inflammatory mediators and promotes the defense against oxidative stress and depresses apoptotic genes in a model of neonatal brain hypoxia/ischemia interpreted as pharmacological preconditioning. We conclude that beta1-adrenoceptor activation might be an efficient strategy for identifying novel pharmacological targets for protection of the neonatal brain.

**Electronic supplementary material:**

The online version of this article (10.1186/s12868-018-0415-2) contains supplementary material, which is available to authorized users.

## Background

During labor at birth, the fetus may experience brain hypoxia–ischemia that may cause severe brain damage and subsequent long-term functional disabilities. Also, during the neonatal period, inflammation and infections may sensitize the immature brain and, subsequent to an episode of hypoxia–ischemia, may aggravate brain damage [1]. In the experimental setting, systemic lipopolysaccharide (LPS)—induced inflammation increases the sensitivity of the neonatal brain to subsequent hypoxic or ischemic events [2, 3]. The perinatal brain experiencing hypoxia–ischemia is also associated with an increase in circulating catecholamines released by the adrenomedullary system, essential for an appropriate cardio-vascular adaptation to hypoxia [4]. The capacity to release catecholamines within the CNS may play a pivotal role in endogenous protection to brain ischemia. For example, lesions in the locus coeruleus caused a decreased noradrenergic input which is associated with aggravated cortical and hippocampal damage following global cerebral ischemia [5]. Conversely, enhanced release of noradrenalin mitigates damage after global cerebral ischemia [6]. Activation of adrenergic receptors downregulate the pro-inflammatory response mediated through a common c-AMP dependent mechanism in several immune cell populations including microglia [7].

We have earlier shown that beta1-adrenoceptor activation provides a robust neuroprotection in the setting of LPS-induced inflammation and oxygen–glucose deprivation (OGD) in a neonatal murine organtypic hippocampal slice culture system [8]. The neuroprotective effect was associated with decreased levels of secreted pro-inflammatory cytokines, including tumor necrosis factor a (TNFα), supporting the notion of an anti-inflammatory action of beta1-adrenoceptor activation. In addition, beta1-adrenoceptor activation was also neuroprotective against OGD without pre-exposure to LPS as well as in hippocampal slices lacking tumor necrosis factor receptor 1 (TNFR1), indicating that beta1-adrenoceptors may activate a broad panel of neuroprotective mechanisms. Earlier studies also have shown that adrenergic stimulation of hippocampal interneurons inhibits excitatory postsynaptic potentials on pyramidal neurons, which may beneficially influence the excitation/inhibition balance during and after cerebral ischemia [9]. Also, astrocytes treated with adrenergic agonists secrete brain-derived neurotrophic factor which could mitigate apoptosis [10].

The hippocampal organotypic slice culture model incorporates parenchymal elements of brain tissue in an intact three-dimensional tissue architecture and thereby it allows studies of the integrated response of all resident cell populations to potentially damaging insults. Taking advantage of this model we have earlier shown that signaling by TNFα through TNFR1 is essential for both LPS-induced sensitization and protection to subsequent in vitro ischemia in the immature murine hippocampus [11]. The aim of this study was to further elucidate the mechanisms of neuroprotection provided by dobutamine by investigating the global expression of mRNA transcripts using the microarray technology.

## Methods

### Preparation and culture of organotypic hippocampal slices

All animal experiments were approved by the Malmö/Lund ethical committee on animal experiments (approval number M 73-04). Hippocampal organotypic tissue cultures were prepared from Balb/c 6-day-old mice (Harlan, Scandinavia, Denmark) according to the method of Stoppini [12] and as described earlier [13]. In brief, mice (n = 120) were deeply anesthesized with 4% isoflurane and decapitated. After removal of brains, hippocampi were dissected in ice-cold Hank’s balanced salt solution containing 20 mmol/L 4-(2-hydroxyethyl)-1-piperazineethanesulfonic acid (HEPES), 100 U penicillin/streptomycin per milliliter, and 3 mg/mL d-glucose and cut into 250 µm thick slices using a McIllwain Tissue Chopper. Slices were plated onto Millicell culture inserts, one slice per insert (0.4 µm Millicell-CM, 12 mm in diameter, Merck Millipore Corp, Bedford, MA, USA), and cultured in an incubator at 35 °C (90–95% humidity, 5% CO_2_) for 9 days. The culture medium consisted of 50% modified Eagle’s medium (MEM), 25% horse serum, and a 18% Hank’s balanced salt solution and was supplemented with 4 mmol/L l-glutamine, 50 units penicillin/streptomycin per milliliter, and 20 mmol/L d-glucose. The pH was adjusted to 7.2 using NaHCO_3_. On day in vitro 1 (DIV1), culture medium was replaced by fresh culture medium. Thereafter, culture medium containing 2% of B27 supplement was changed three times during the first week of cultivation. After this period, B27 supplement was omitted and replaced by the same volume of water. On DIV9, when the experiment was initiated, horse serum was omitted and the volume was replaced by MEM. This medium was used throughout the subsequent experiments. All substances were obtained from Life Technologies (Carlsbad, CA, USA), d-glucose from Sigma-Aldrich (St Louis, MO, USA).

### Oxygen–glucose-deprivation (OGD)

Cultures were washed once in phosphate-buffered saline (PBS) and transferred to an anaerobic incubator (10% H_2_, 5% CO_2_, and 85% N_2_, temperature 35 ± 0.3 °C) (Elektrotek Ltd, Keighley, UK) according to previously described protocols [13]. Inside the incubator, culture inserts containing the slices were transferred to wells containing pre-equilibrated OGD medium (concentrations in mmol/L: 2 CaCl_2_, 125 NaCl, 25 NaHCO_3_, 2.5 KCl, 1.25 NaH_2_PO_4_, 2 MgSO_4_, and 10 sucrose, pH 7.4). After 15 min, the slices were transferred to normoxic fresh culture medium and placed in a CO_2_ incubator.

### Experimental protocol

All cultures used in one experiment were prepared from pups from one to two females with litters born at the same day. Experiments were started on DIV9, and slices were assorted into four experimental groups with one slice from each individual mouse per experimental group and six slices per group on one culture plate. Light microscopy was used to include only slices with an organotypic morphology.

The experimental groups were assigned tofollow the above mentioned paradigms: (1) control—cultures exposed to control medium during 4 h; LPS—cultures exposed to LPS (1 µg/mL; Sigma-Aldrich) for 4 h; D (dobutamine)—cultures exposed to dobutamine (50 µM, Sigma-Aldrich) for 4 h; LPS/D—cultures exposed to LPS (1 µg/ml) + D (50 µM) for 4 h and (2) LPS/OGD—incubation with LPS for 24 h followed by oxygen–glucose deprivation (OGD) and thereafter transfer to fresh medium for 2 h; OGD—pre-incubation without LPS for 24 h, followed by OGD and thereafter transfer to fresh medium for 2 h; D/OGD—pre-incubation with dobutamine for 24 h, followed by OGD and thereafter transfer to fresh medium for 2 h; D/LPS/OGD—incubation with dobutamine + LPS for 24 h followed by OGD and transfer to fresh medium for 2 h. At the end of each experimental protocol, slices were snap-frozen on dry ice and stored at − 70 °C until further processing. Slice cultures to quantify SOD3 levels were harvested 24 h after OGD.

**Fig. 1:**
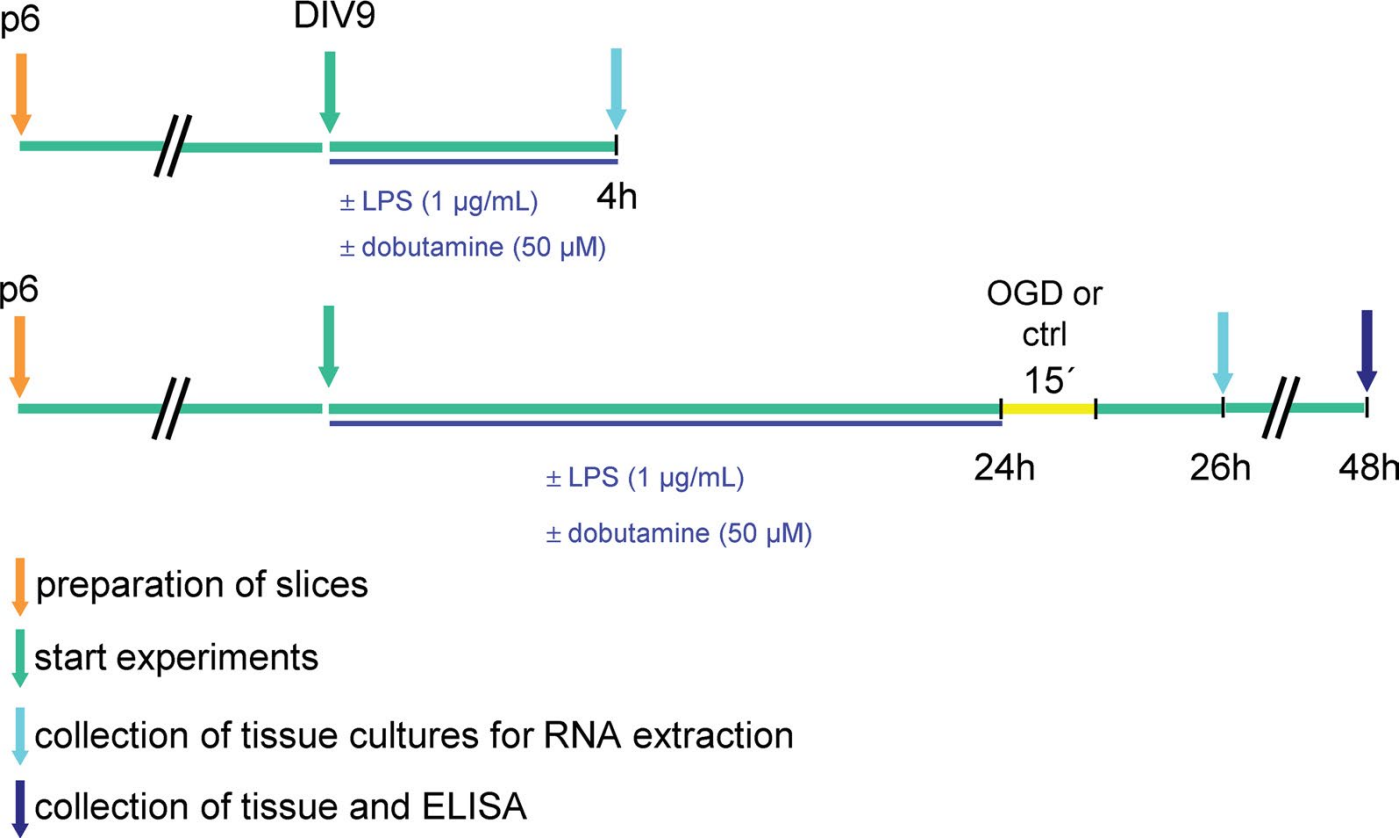
Experimental design. Hippocampal slices were prepared from postnatal day 6 and grown for 9 days in vitro. Tissue was collected (arrows) and snap frozen for RNA transcript analysis at 4 h of incubation with either lipopolysaccharide (LPS, 1 µg/ml)) or dobutamine or a combination thereof (paradigm 1). Alternatively, slices were incubated with either lipopolysaccharide (LPS, 1 µg/ml)) or dobutamine or a combination thereof for 24 h and thereafter incubated under conditions of oxygen–glucose deprivation (OGD) for 15 min with an additional 2 h of recovery in normoxic and normoglycemic conditions. Twentyfour hours after stimulation (normoxic control or OGD), protein levels of SOD3 were determined by ELISA (paradigm 2)

Each experimental group, as described above, consisted of 4 samples. Each sample included pooled RNA (in total 5–12 mg) from 12 slices from 12 individual mice. An overview of the experimental design is given in Fig. 1.

### Analysis of mRNA transcript

Oligo nucleotide microarrays were produced at the SCIBLU Genomics Center, Lund University, Sweden (www.lth.se/sciblu) using a set of ~ 37,000 mouse oligonucleotide probes (Operon Ver. 4.0) as previously described [14]. Total RNA was extracted from 10 to 15 mg frozen tissue, using RNeasy lipid tissue mini kit (Qiagen, Valencia, CA, USA) and quality controlled using a BioAnalyzer 2100 system (Agilent Technologies, Kista Sweden). The Universal Mouse reference RNA (Stratagene, La Jolla, CA, USA) was used as reference in all experiments. Fluorescently labeled cDNA targets for hybridization were prepared according to manufacturers’ instructions using the Corning Pronto Plus System 6 (Corning Life Sciences, NY, USA). Samples were labeled with Cy3-dCTP (Amersham) and reference was labeled with Cy5-dCTP. Hybridization was performed overnight at 42 °C using a MAUI hybridization system (BioMicro Systems, Salt Lake City, USA), and slides were washed according to the Corning Pronto Plus system instructions. Fluorescence was recorded using an Agilent G2565AA microarray scanner (Agilent Technologies).

### Image and data analysis

Tagged Image File Format images were analyzed using the GenePix Pro 4.0 software (Axon Instruments, Foster City, CA), and the quantified data matrix was loaded into a BioArraySoftware Environment (BASE) [15]. Positive and non-saturated spots were background corrected using the median foreground minus the median background signal intensity for each channel. Data was filtered for flagged features from the image analysis, features with signal to noise (SNR) < 2 in both channels and features without a known gene symbol prior to “lowess” normalization [16]. Reporters not represented at least one time per experiment group were removed (equaling 92% presence required). Differentially expressed genes between experimental groups were identified using a *t* test with a false discovery rate (FDR) of 5%. Quantitative differences between experimental groups were expressed as log2 fold changes, ratio of mean intensities of the respective groups.

The primary objective of data analysis was to evaluate the effect of incubation with the β1-adrenergic agonist dobutamine on the different experimental paradigms, i.e. on control conditions, on the LPS-induced pro-inflammatory response, on sole OGD and finally on LPS-induced sensitization to OGD. Thus, comparisons between the groups were performed as follows: D versus Control; D/LPS versus LPS; D/OGD versus OGD; D/LPS/OGD versus LPS/OGD. Quantitative differences in gene expression in paired comparisons were analysed as described above. To assess particular effect of treatment we considered genes that were changed at least two-fold between two experimental conditions. The lists of these genes are displayed in Additional files 1, 2, 3 and 4: Tables S1 to S4.

### Real-time PCR

Total RNA was extracted from frozen tissue using Trizol^®^ (GIBCO BRL) according to the manufacturer’s instructions and performed as essentially described previously [17]. For cDNA synthesis RNA (0.4 μg) was reverse-transcribed in a final volume of 20 μL using reverse transcription reagents (Applied Biosystems, Roche, Nutley, New Jersey) according to the manufacturer’s protocols. The samples were stored at − 20 °C until further use. Gene transcripts were quantified using real-time PCR on ABI PRISM^®^ 7000 sequence detection system (Applied Biosystems). Primers and probes were ordered from Assays by-Design TM (Applied Biosystems). PCR reactions were assayed in a 25 μL final volume containing final concentrations: 1× TaqMan Universal Master Mix (Applied Biosystems), 1× Assaymix (Applied Biosystems) and 1.6 μL of 20 ng/μL of a DNA aliquot. The thermal cycling conditions were initiated by UNG activation at 50 °C for 2 min and an initial denaturation at 95 °C for 10 min, followed by 40 cycles of denaturation at 95 °C for 15 s, annealing and extension at 60 °C for 1 min. Two negative controls, with vehicle, were included for every probe that was run at one occasion. Each reaction was assayed in duplicate. A calibration curve, obtained by serial four-fold dilutions of the template DNA (0.08–80 ng), was used to quantify each sample. The quantitative value of each sample was normalized to the corresponding value of *Gapdh* and results expressed as relative values.

### Superoxid dismutase 3 enzyme-linked immunosorbent assay (ELISA)

Levels of SOD3 were measured by ELISA according to manufacturers instructions (Uscn Life Science Inc. Wuhan, China). In brief, slices were homogenized in lysis buffer and protein concentration has been determined by the Bradford assay [18]. Lysates (100 µl) were added to the assay plate and incubated at 37 °C for 2 h. Thereafter, samples were consecutively incubated with solutions A, solution B, substrate and stop solution with washing steps in between (3 × 2 min each). Finally, the plate was read at 450 nm using a microplate reader (Biorad model 860) and enzyme activities were calculated against the standards provided with the assay and normalized to the protein concentration.

### Statistics

Data are expressed as mean ± standard error of mean (SEM). Statistical analyses have been performed with GraphPad Prism 6 software (GraphPad, San Diego, CA, USA) by using ANOVA followed by Bonferroni multiple comparison test with p < 0.05 considered to represent statistical significance.

## Results

### The effect of dobutamine on gene expression

In naive control slice cultures treated with a neuroprotective dose of dobutamine, 220 genes were up-regulated and 78 were down-regulated. A specific effect of dobutamine treatment was the up-regulation of genes coding enzymes involved in the defense against oxidative damage, i.e. *superoxide dismutase 3* (*Sod3*), *coenzyme Q10 homolog B, sulfiredoxin 1, sestrin 2, thioredoxin reductase 1* (Table 1). Importantly, genes coding for anti-inflammatory proteins, such as *colony stimulating factor 2 receptor* and *CD14* were also up-regulated, while a large group of pro-inflammatory mediators, such as *Ccl3, Cxcl10, Ifit2,* and *Iigp1*, were down-regulated (Table 2). In addition, a general feature of the gene expression profile was a dominant upregulation of genes associated with signalling and transcription (Additional files 1 and 2: Tables S1 and S2). Evidently, dobutamine treatment activated genes with a clear protective and anti-inflammatory profile.

**Table 1:** Regulated genes involved in oxidative stress in all experimental groups classified by gene ontology

Genes related to oxidative stress	Ctrl versus Ctrl D	Ctrl versus LPS	Ctrl versus OGD	OGD versus OGD D	LPS versus LPS D	LPS OGD versus LPS D OGD
Aco2, Aconitase 2, mitochondrial	3.6					
Txndc11, Thioredoxin domain containing 11	3.2				2.8	
Oxnad1, Oxidoreductase NAD-binding domain containing 1	3.0					
Coq10b, Coenzyme Q10 homolog B (S, cerevisiae)	2.6					
Sod3, Superoxide dismutase 3, extracellular	2.5			9.6		3.2
Srxn1, Sulfiredoxin 1 homolog (S, cerevisiae)	2.5				2.4	
Txnrd1, Thioredoxin reductase 1	2.3					
Ncf2, Neutrophil cytosolic factor 2	2.1					
Sesn2, Sestrin 2	2.1					
Vav1, Vav 1 oncogene	0.5					
Cybb, Cytochrome b-245, beta polypeptide	0.4	7.6			0.5	
Ncf1, Neutrophil cytosolic factor 1	0.4					
Syk, Spleen tyrosine kinase		2.9				
Nfe2l1, Nuclear factor, erythroid derived 2,-like 1		2.4				
Sod2, Superoxide dismutase 2, mitochondrial		2.3				
Naprt1, Nicotinate phosphoribosyltransferase domain containing 1		0.5				
Txnip, Thioredoxin interacting protein		0.5				
Sesn1, Sestrin 1		0.4				
Nxn, Nucleoredoxin		2.2				
Hmox1, Heme oxygenase 1			0.2		0.4	
Oxnad1. Oxidoreductase NAD-binding domain containing 1					3.1	
Hspa5. Heat shock 70 kD protein 5					2.5	
Hspb1. Heat shock protein 1					2.1	
Ncf1. Neutrophil cytosolic factor 1					0.5	
Cdo1, Cysteine dioxygenase 1, cytosolic						2.1
Xdh, Xanthine dehydrogenase						0.5
Gstt1, Glutathione S-transferase, theta 1						0.4

*D* dobutamine, *LPS* lipopolysaccharide, *OGD* oxygen–glucose-deprivation

**Table 2:** Regulated genes involved in inflammation in all experimental groups classified by gene ontology

Inflammation related genes	Ctrl versus Ctrl D	Ctrl versus LPS	Ctrl versus OGD	OGD versus OGD D	LPS versus LPS D	LPS OGD versus LPS D OGD
Ptgs2, Prostaglandin-endoperoxide synthase 2	11.9	42.8				0.4
Csf2rb1, Colony stimulating factor 2 receptor, beta 1	8.7					
Cd14, CD14 antigen	8.4				2.7	
Tgm2, Transglutaminase 2, C polypeptide	7.2	7.4				0.4
Bcl3, B-cell leukemia/lymphoma 3	6.9					
Socs3, Suppressor of cytokine signaling 3	5.8	11.7	2.6			
Cd86, CD86 antigen	4.2		2.2			
Sbno2, Strawberry notch homolog 2 (Drosophila)	3.4					
Clcf1, Cardiotrophin-like cytokine factor 1	3.2					
Litaf, LPS-induced TN factor	3.0	2.7				
Edg3, Endothelial differentiation, sphingolipid G-protein-coupled receptor, 3	2.9					
Fcgr3, Fc receptor, IgG, low affinity III	2.6					
Osmr, Oncostatin M receptor	2.5	5.1				
C5ar1, Complement component 5a receptor 1	2.4					
Il4ra, Interleukin 4 receptor, alpha	2.4				3.1	
Pde4d, Phosphodiesterase 4D, cAMP specific	2.4					
Ccrl2, Chemokine (C–C motif) receptor-like 2	2.3	62.3				0.3
P2rx7, Purinergic receptor P2X, ligand-gated ion channel, 7	2.3					
Tnfrsf1b, Tumor necrosis factor receptor superfamily, member 1b	2.3	5.0				0.4
Cxcr7, Chemokine (C-X-C motif) receptor 7	2.2				3.6	
Tgfbi, Transforming growth factor, beta induced	2.1					
Fbxl8, F-box and leucine-rich repeat protein 8	0.5					
Iigp1, Interferon inducible GTPase 1	0.5	131.2			0.4	0.1
March1, Membrane-associated ring finger (C3HC4) 1	0.5					
Peli2, Pellino 2	0.5	0.4				
Prrx1, Paired related homeobox 1	0.5					
Cmtm7, CKLF-like MARVEL transmembrane domain containing 7	0.4					
Ifit2, Interferon-induced protein with tetratricopeptide repeats 2	0.4	20.5			0.3	0.1
Pilra, Paired immunoglobin-like type 2 receptor alpha	0.4					2.3
Selpl, Selectin, platelet (p-selectin) ligand	0.4					
Ccl3, Chemokine (C–C motif) ligand 3	0.3	280.8			0.1	
Cx3cr1, Chemokine (C-X3-C) receptor 1	0.3				0.4	
F3, Coagulation factor III	0.3					
Cxcl10, Chemokine (C-X-C motif) ligand 10	0.2	126.4		2.5		0.3
Tnf, Tumor necrosis factor		625.9	3.2		0.1	
Ifit1, Interferon-induced protein with tetratricopeptide repeats 1		143.8				0.3
Mpa2 l, Macrophage activation 2 like		120.1			0.5	0.3
Tnfaip3, Tumor necrosis factor, alpha-induced protein 3		111.8			0.4	0.5
Tnfaip2, Tumor necrosis factor, alpha-induced protein 2		102.3				0.4
Igtp, Interferon gamma induced GTPase		96.0			0.4	0.2
Irf7, Interferon regulatory factor 7		73.2				0.2
Tyki, Thymidylate kinase family LPS-inducible member		72.0			0.4	0.1
Ptx3, Pentraxin related gene		60.0				
Slfn5, Schlafen 5		37.9				0.4
Slfn2, Schlafen 2		37.2				0.5
Irf1, Interferon regulatory factor 1		37.1		2.3	0.4	0.5
Tap1, Transporter 1, ATP-binding cassette, sub-family B (MDR/TAP)		36.3				
Tlr2, Toll-like receptor 2		32.0				
Ripk2, Receptor (TNFRSF)-interacting serine-threonine kinase 2		23.9				
Slfn9, Schlafen 9		21.4				0.3
Ly6a, Lymphocyte antigen 6 complex, locus A		16.4				0.1
Ifi35, Interferon-induced protein 35		16.1				0.3
Ifi202b, Interferon activated gene 202B		14.1				0.4
Tap2, Transporter 2, ATP-binding cassette, sub-family B (MDR/TAP)		13.0				0.4
Samhd1, SAM domain and HD domain, 1		11.4				0.4
Myd88, Myeloid differentiation primary response gene 88		9.6				
Ifi44, Interferon-induced protein 44		8.8				0.3
Plaur, Plasminogen activator, urokinase receptor		7.5				
Tnip1, TNFAIP3 interacting protein 1		7.5				
Rnf125, Ring finger protein 125		7.1				
Ifi203, Interferon activated gene 203		5.8				0.3
H2-M3, Histocompatibility 2, M region locus 3		5.7				0.4
Nmi, N-myc (and STAT) interactor		5.0				0.4
Irak2, Interleukin-1 receptor-associated kinase 2		4.7				
Tapbp, TAP binding protein		4.5				0.4
Il17ra, Interleukin 17 receptor A		4.4				
Tapbpl, TAP binding protein-like		4.2				
Tcirg1, T-cell, immune regulator 1		3.7				
Plek, Pleckstrin		3.3				
Irf2, Interferon regulatory factor 2		3.2				
Plau, Plasminogen activator, urokinase		3.1				
Ifitm3, Interferon induced transmembrane protein 3		2.9				0.4
Ifrg15, Interferon alpha responsive gene		2.9				
Tnfrsf14, Tumor necrosis factor receptor superfamily, member 14 (herpesvirus entry mediator)		2.8				0.4
Irf5, Interferon regulatory factor 5		2.7				
Tnfrsf12a, Tumor necrosis factor receptor superfamily, member 12a		2.7				
C3, Complement component 3		2.5		2.2		
Ifitm1, Interferon induced transmembrane protein 1		2.5				
Il18 bp, Interleukin 18 binding protein		2.5				0.4
Tcrb-V13, T-cell receptor beta, variable 13		2.4				
Traf2, Tnf receptor-associated factor 2		2.4				
Cxcl12, Chemokine (C-X-C motif) ligand 12		2.3				
Traf5, Tnf receptor-associated factor 5		2.3				0.5
Lcp1, Lymphocyte cytosolic protein 1		2.2				
Socs2, Suppressor of cytokine signaling 2		2.2				
Igsf10, Immunoglobulin superfamily, member 10		2.1				
Ptgir, Prostaglandin I receptor (IP)		2.1				
Peli1, Pellino 1		2.0				
Tnfsf12, Tumor necrosis factor (ligand) superfamily, member 12		0.5				
Ptgs1, Prostaglandin-endoperoxide synthase 1		0.4				
Ptgds2, Prostaglandin D2 synthase 2, hematopoietic		0.3				
Cxcr4, Chemokine (C-X-C motif) receptor 4		0.2				
Hmha1, Histocompatibility (minor) HA-1		0.1				
Ccl4, Chemokine (C–C motif) ligand 4		329.0			0.3	0.4
Trem2, Triggering receptor expressed on myeloid cells 2		0.4				2.5
Lgals3, Lectin, galactose binding, soluble 3			3.5			0.3
Aebp1 (gUuMeU):AE binding protein 1			2.2			
Trib1, Tribbles homolog 1 (Drosophila)			2.2			0.5
Gbp6, Guanylate binding protein 6				2.7		0.4
Cd72, CD72 antigen				0.5		
Inhba. Inhibin beta-A					5.4	
Dusp14. Dual specificity phosphatase 14					2.6	
Itga1. Integrin alpha 1					2.6	
Csrp2. Cysteine and glycine-rich protein 2					2.5	
Csrp2. Cysteine and glycine-rich protein 2					2.4	
Ampd3. AMP deaminase 3					0.5	
C1r. Complement component 1. r subcomponent					0.5	0.3
C3ar1. Complement component 3a receptor 1					0.5	
Cd274. CD274 antigen					0.5	0.5
H2-D1. Histocompatibility 2. D region locus 1					0.5	0.1
Tgfb3. Transforming growth factor. beta 3					0.5	
Tgfbr2. Transforming growth factor. beta receptor II					0.5	
Tlr4. Toll-like receptor 4					0.5	
Gvin1. GTPase. very large interferon inducible 1					0.4	0.3
Csf1. Colony stimulating factor 1					0.3	0.4
S100a4, S100 calcium binding protein A4						2.1
Csf1r, Colony stimulating factor 1 receptor						2.0
Fcgr2b, Fc receptor, IgG, low affinity IIb						0.5
Scarf2, Scavenger receptor class F, member 2						0.5
Stat2, Signal transducer and activator of transcription 2						0.5
Gimap5, GTPase, IMAP family member 5						0.4
H2-D4, Histocompatibility 2, D region locus 4						0.4
H2-K1, Histocompatibility 2, K1, K region						0.4
Trafd1, TRAF type zinc finger domain containing 1						0.4
Trex1, Three prime repair exonuclease 1						0.4
B2 m, Beta-2 microglobulin						0.3
Casp1, Caspase 1						0.3
Fcgr1, Fc receptor, IgG, high affinity I						0.3
Irgm, Immunity-related GTPase family, M						0.3
Stat1, Signal transducer and activator of transcription 1						0.3
Bst2, Bone marrow stromal cell antigen 2						0.1
Ly6c, Lymphocyte antigen 6 complex, locus C						0.1
Ly6e, Lymphocyte antigen 6 complex, locus E						0.1

*D* dobutamine, *LPS* lipopolysaccharide, *OGD* oxygen–glucose-deprivation

### The effect of pretreatment with dobutamine on LPS induced gene expression

Combining LPS exposure with dobutamine treatment was characterized by a lower number of genes significantly regulated and an up-regulation of genes coding for the anti-apoptotic and survival proteins, Bag3, Tmbim1, Tinf2, for proteins of the defense against oxidative damage as *Txndc11* (Thioredoxin domain containing 11), *Srxn1*, (Sulfiredoxin 1 homolog (S, cerevisiae)), *Oxnad1* (Oxidoreductase NAD-binding domain containing 1), *Hspa5* (Heat shock 70 kD protein 5) and *Hspb1* (Heat shock protein 1) and anabolic biosynthetic pathways (Table 1 and Additional file 3: Table S3). However, the most prominent change was a strong down-regulation of pro-inflammatory mediators such as *Tnf, Ifit2, Irf1, Iigp1, complement component C1r, complement component C3ar1* (Table 2), genes involved in generating oxidative stress, *neutrophil cytosolic factor 1,* and *cytochrome b*-*245* (Table 1). Clearly, dobutamine pretreatment preconditioned the slices to a subsequent challange with the highly pro-inflammatory agent LPS.

### Effect of LPS treament on gene expression

LPS-exposure also induced massive gene regulation, Table 1 and Supplement II. After 4 h of exposure, 389 genes were up-regulated and 120 were down-regulated in the hippocampal slices. Particularly, there was the up-regulation of 64 inflammation associated genes (Table 2) and genes associated with signalling and tracription (Additional files 1 and 2: Tables S1 and S2). *TNFa*, genes coding for several TNF-induced inflammatory mediators, and several interferon induced genes were up-regulated, such as *interferon*-*induced protein with tetratricopeptide repeats 1 (Ifit1), interferon inducible GTPase 1 (Iigp1), Ifit2, Irf1* and *Irf7*. Genes regulating signalling downstream from cytokine receptors were also strongly up-regulated: the Nuclear factor of kappa light chain gene enhancer in B-cells *(NFκB)* signaling pathway, (*Nfkb1, Nfkb2, Nfkb inhibitor (Nfkbi) beta, Nfkbi alpha, Nfkbi epsilon* and *Nfkbi zeta) and*, the JAK/STAT pathway (*Signal transducer and activator of transcription 1 (Stat1), Stat2 and Stat3)*. The expression profile demonstrates a strong induction of inflammatory mediators and signalling provoked by LPS in the hippocampal slices.

### Gene expression after oxygen/glucose deprivation (OGD)

Comapred to the LPS and dobutamine treated slices, a modest regulation of genes was seen in slices subjected to 15 min of OGD, with 41 genes up-regulated and 26 down-regulated at 2 h after OGD. Still, the gene expression profile showed almost half of the > two-fold up-regulated genes being transcriptional regulators, mostly transcription factors and notably *FBJ osteosarcoma oncogene B (FosB),* and *nuclear receptor subfamily 4.* This strong postischemic induction of these immediate early genes has earlier been described in models of transient ischemia in the neonatal or adult rat brains [17, 19], where induction of *brain derived neurotrophic factor,* and signaling molecules such as *dual specific phosphatates*-*4* were reported. In addition, inflammatory mediators such as *galectin*-*3* and *tumor necrosis factor (Tnf)* are prominently induced after OGD, as are the protective growth factors *BDNF, Hbegf, Heparin*-*binding EGF*-*like growth factor*, and *Glial cell line derived neurotrophic factor family receptor*. Hence, within 2 h after OGD gene transcription is dominated by immediate early gene expression, with a rapid induction of several genes coding for protective growth factors (Additional file 3: Table S3).

### The effect of pretreatment with dobutamine prior to OGD

When slices were pretreated with a neuroprotective concentration of dobutamine prior to OGD, only 14 genes were additionally regulated as compared to OGD alone, and when measured at 2 h after the OGD insult. The most prominent change was an almost ten-fold activation of the extracellular *superoxide dismutase (Sod3*) gene and *Nupr1* a cell survival gene (Table 1 and Additional file 3: Table S3).

### The effect of pretreatment with dobutamine on the LPS/OGD induced gene expression

Pretreatment of hippocampal slices with dobutamine in combination with LPS 24 h prior to OGD induced a remarkable strong down-regulation of 136 genes and only 20 genes were up-regulated at 2 h after the insult, compared to LPS/OGD treated slices, (Tables 1 and 2, Additional files 1, 2, 3, 4, 5, 6 and 7: Tables S1 to S7). Again, the *Sod3* gene was upregulated several fold. Among the down-regulated genes, 55 were related to inflammation, particularly several interferon regulated genes, such as *Ifit2, Iigp1, Irf7, Igtp, Ifit1, Interferon*-*induced protein 35 (Ifi35), Ifi44, Interferon activated gene 202B (Ifi202b), Ifi203* and *Nfkbi epsilon* and *C1r*. Also, several chemokine related genes were downregulated such as, *Ccrl2, Cxcl10, Cxcl12,* and *Ccl4*.

### Genes regulated by dobutamine treatment in multiple experimental paradigms

To further narrow down the number of genes relevant for the neuro-protective effect of dobutamine, we determined common genes regulated by dobutamine treatment in at least two experimental comparisons. A prominent feature is the upregulation of the thioredoxin reductase as well as *superoxide dismutase 3,* the latter induced several-fold in three different conditions: dobutamine only, dobutamine + OGD, dobutamine + OGD/LPS. Also *Bag3, Inhibin B, Tinf2 and TMBIM* are all related to anti-apoptotic and survival promoting proteins. Furthermore, dobutamine supports anabolic processes including carbohydrate and lipid metabolism. In particular *solute carrier family 16, member 3*, or monocarboxylate transporter 4 (MCT4), and *phosphoglucomutase 5* were up-regulated in multiple paradigms. The strong anti-inflammatory action of dobutamine was evident by the vast number of downregulated genes associated with inflammatory processes in several experimental paradigms. Notably, *CD72* and three interferone-inducible genes were down-regulated in three different dobutamine treatment combinations (Additional files 1–7: Tables S1 to S7).

### Verification of expression patterns

**Fig. 2:**
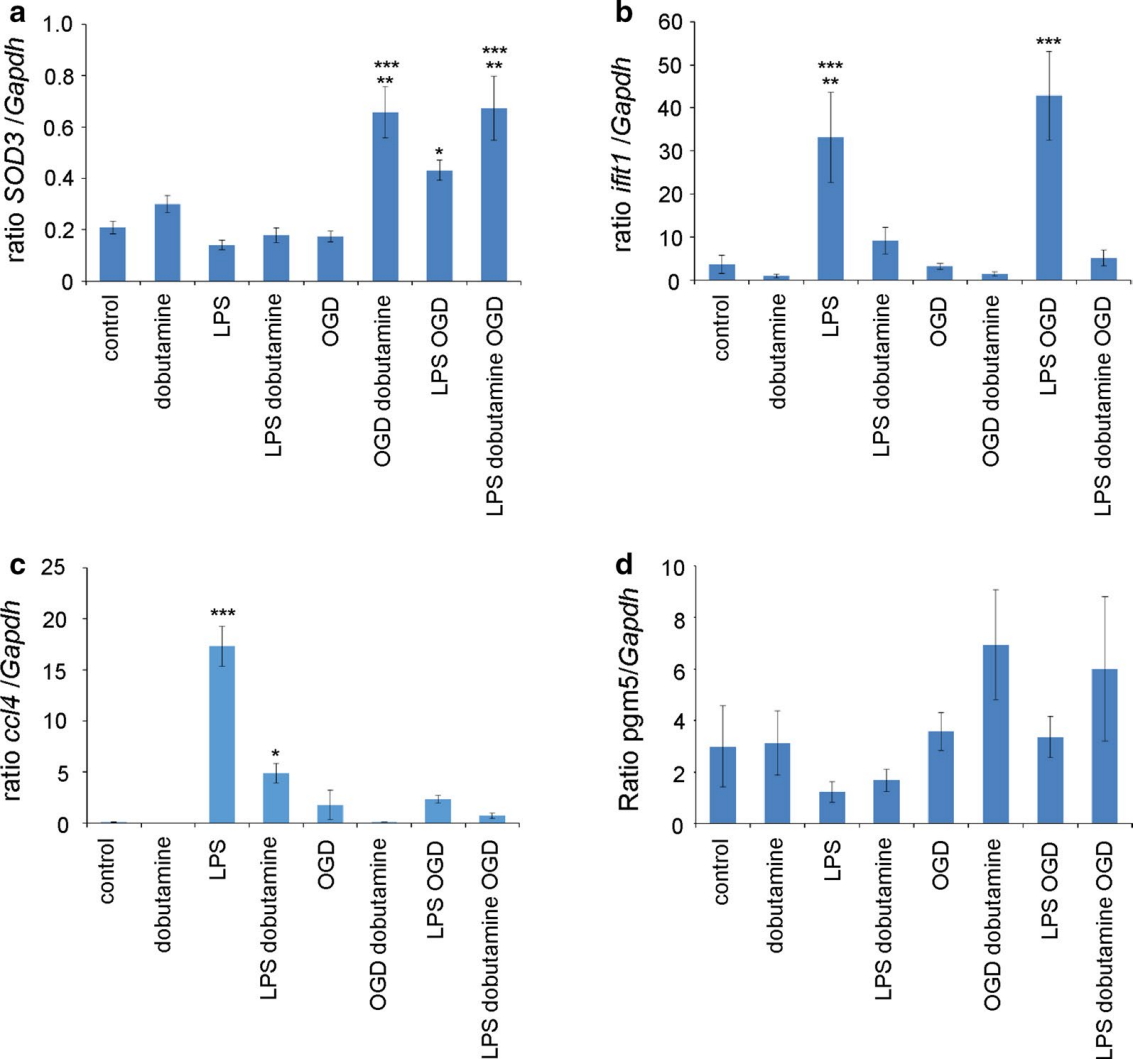
Expression levels of genes determined by qPCR. To validate the findings of the micoarray the expression levels of a gene downregulated by dobutamine treatment, expression of **a** *Sod3,* **b** *Ifit2,* **c** *ccl4* and **d** *pgm5* were measured by qPCR. Levels are normalized *Gapdh*. Data are shown as mean ± SEM, with n = 14 (OGD); n = 9 (LPS + OGD); n = 9 (LPS + Dobutamine + OGD); n = 10 (Control); n = 7 (LPS); n = 8 (Dobutamine + LPS); n = 6 (Dobutamine + OGD); n = 8 (Dobutamine). Differences were assessed as described in the Methods section for independent samples between all experimental groups. The following differences were obtained: *p < 0.05, **p < 0.001, ***p < 0.0001. **a** *Sod3:* p < 0.0001 OGD dobutamine versus control, versus LPS, versus LPS dobutamine and versus OGD; LPS dobutamine OGD versus control, versus LPS, versus LPS dobutamine and versus OGD. p < 0.001 OGD dobutamine versus dobutamine; LPS dobutamine OGD versus dobutamine. p < 0.05 OGD versus LPS OGD. **b** *Ifit1*: p < 0.0001 LPS versus OGD; LPS OGD versus control, versus LPS dobutamine, dobutamine, OGD, OGD dobutamine, LPS dobutamine OGD; p < 0.001 LPS versus, control, versus dobutamine, versus OGD dobutamine, versus LPS dobutamine OGD. **c** *Ccl4:* p < 0.0001 LPS versus all other experimental groups; p < 0.05 LPS dobutamine versus control. **d** *Pgm5:* No significant differences have been obtained (p < 0.18)

In order to assess the relevance of the microarray data, the expression levels of four genes of different gene classes were measured by qPCR, Fig. 2. In analogy with the microarray results, dobutamine significant depressed *Ifit2* expression in multiple experimental paradigm and increased the levels of *sod3*. The microarray data were further validated by analyzing the correlation between expression levels measured by qPCR. There was a higly significant correlation in all analyzed genes, *Ifit2* (r = 0.941, p < 0.001), *Sod3* (r = 0.956, p < 0.001), *Pgm5* (r = 0.563, p < 0.001), *ccl4* (r = 0.982, p < 0.001).

**Fig. 3:**
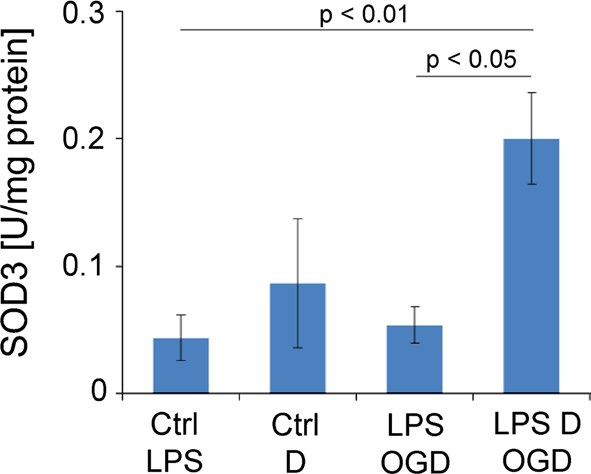
Protein levels of SOD3. Levels of SOD3 determined by ELISA in hippocampal sclice cultures 24 h following OGD. Results are presented as units normalized to one milligram of protein. Experiments have been carried out from two independent experiments including at least six individual slice cultures. Statistical differences have been calculated by one-way ANOVA followed by Bonferroni posthoc correction and are displayed in the figure. Intra-assays cv’s: Ctrl LPS—0.7 to 29.9%, Ctrl D—1.7 to 11.6%, LPS OGD 0 to 14.6%, LPS D OGD 0.7 to 16.1%; inter-assays cv’s: Ctrl LPS—11.6%, Ctrl D—6.8%, LPS OGD 5.0%, LPS D OGD 5.4%

In addition, we performed ELISA for SOD3. As shown in Fig. 3, levels of SOD3 significantly increased in slice cultures exposed to OGD and pretreated with LPS and dobutamine (0.2 ± 0.03 U/mg protein) compared cultures exposed to OGD and preconditioned only with LPS (0.054 ± 0.01 U/mg protein). Likewise, control cultures treated with dobutamine showed slightly increased levels of SOD3 (0.087 ± 0.05 U/mg protein) compared to untreated control cultures (0.04 ± 0.01 U/mg protein), although no statistical differences have been reached comparing the two control conditions.

## Discussion

The neuroprotective actions of dobutamine against damage induced by OGD and LPS/OGD [8, 11] probably reside in activation of multiple parallell and synergistic processes, such as up-regulation of defense systems or down-regulation of detrimental mechanisms triggered by the hypoxic/aglycemic insult alone or in combination with an inflammatory surge [19]. The dobutamine activated processes, may either protect the slices by preconditioning against the subsequent OGD insult or boost protective mechanisms during reoxygenation after OGD. From the gene ontology analysis, derived from comparisons among different treatment paradigms, we see clear evidence of a dobutamine induced regulation of genes associated with the inflammatory process, and regulation of genes promoting survival and associated with the defense against oxidative stress.

### Hypoxia/aglycemia and LPS-induced gene expression

In the two paradigms we have studied slices exposed to either to hypoxia/aglycemia or LPS. The gene expression profile is similar to that seen in similar conditions in vivo [20, 21]. Hence, compared to the control group, OGD markedly induced immediate early genes such transcription factors (*Fosb, Nr4a, Maff, Erg1, Erg2)*, signaling molecules *DUSP* and others such as *Homer 1* and *bdnf* as demonstrated earlier in rat models of brain ischemia [20].

Likewise, stimulation of hippocampal slices with LPS upregulated genes involved in the TLR-4 mediated innate immune response. The groups of genes included, *TNFα* and TNF induced genes, toll like receptor signaling including the regulator MyD88, genes involved in MAP kinase and JAK/STAT signaling, activation of NF-κB, a large group of interferon-induced genes and genes involved in the remodeling of the cytoskeleton [22]. In our model of LPS-induced inflammation both the MyD88 dependent and the MyD88 independent pathway were engaged, which is supported by similar studies performed in cultured macrophages [23]. The MyD88-dependent pathway is critical for the expression of inflammatory cytokines, whereas the MyD88-independent pathway induces expression of genes containing IFN-stimulated regulatory elements resulting in the expression of *Ip*-*10 (Cxcl10)* [24]. MyD88-independent pathway is propagated by an initial activation of members of the interferon regulatory factor (IRF) family of inflammatory mediators. The IRFs in turn activate the JAK/STAT pathway [25]. In our data set, several interferon regulated factors were up-regulated, including, *Ifit, Iigp1, Irf1* and *Irf7*, as were members of the JAK/STAT signaling pathway and the effector *Ip*-*10 (Cxcl10)*. The observed up-regulation of *Tlr*-*2* has been shown to depend on IRF activation following *Tlr*-*4* stimulation [25, 26].

Stimulation of slice cultures with LPS induced a number of genes coding for effector proteins that activate the function of immune cells and coordinate various effector cells of innate immunity in the brain. These included the chemokines Macrophage inflammatory protein-1β *(Ccl4/Mip*-*1β)*, a chemoattractant for NK-cells and monocytes [27], macrophage inflammatory protein-1α *(Ccl3/Mip*-*1α)* which is involved in the recruitment and activation of polymorphonuclear leukocytes and has been shown to be induced by LPS treatment in fetal microglia [28], *Cxcl10*, which may induce neuronal apoptosis [29] and the chemokine receptor *Ccrl2*. In addition, several genes coding for adhesion molecules such as *Vcam*-*1* genes coding for MHC II, as well as genes controlling its activity, were up-regulated and in cooperation with the mentioned chemokines aid in leukocyte extravasation. The gene coding for *Tnfα* exhibited the highest fold up-regulation of all genes following 4 h of LPS exposure whereas genes coding for other pro-inflammatory cytokines were not significantly regulated. The gene coding for *Ifit 1* was highly up-regulated, recently described as a novel marker of microglial activation in LPS-exposed microglia in vitro, as in the hippocampus in vivo [22]. Taken together, LPS induces a strong gene response with all hallmarks of a pro-inflammatory state.

### The role of beta1-adrenoceptor activation on inflammation and exidative stress related genes

The protective action of beta1-adrenoceptor activation by dobutamine has recently been associated with expression of heat-shock protein 70 in lymphoma cell line [30]. We show here that in brain slices this is clearly associated with changes in gene expression including depression of pro-inflammatory genes and upregulation of genes associated with defense of oxidative stress. LPS exposure and OGD stimulation per se are considered as two different experimental paradigms. Hence, in both groups of genes are coding for proteins involved in preconditioning mechanisms to protect cells, i.e. neurons, against an otherwise lethal ischemic/hypoxic stimulus. It seems, therefore, be likely that dobutamine modulates different genes dependent on the stimulus finally resulting in protection of cells following ischemia.

While dobutamnine upregulated some inflammatory genes in control cultures (Table 2), the LPS induced activation of inflammatory genes was strongly attenuated by co-activation of the beta1-adrenoceptor, including the intra-cellular pathways downstream of *Tlr*-*4* activation and of effector proteins and molecules associated with antigen presentation and immune cell recruitment as well as effector proteins involved in immune cell recruitment. The highest fold-change of down-regulated genes was observed for *Tnfα*, which is in line with our previous finding of decreased levels of secreted TNFα by beta1-adrenoceptor stimulation during LPS exposure [11]. Signaling by *Tnfα* through *Tnfr1* is essential for the aggravated neuronal cell death caused by LPS-pre-exposure immediately prior to OGD [8]. The down-regulation of *Tnfα* transcription by beta1-adrenoceptor stimulation is most probably one critical mechanism whereby beta1-adrenoceptor stimulation offers neuroprotection.

Pre-treatment with dobutamine had also a profound down-regulatory effect on pro-inflammatory response following the combined LPS/OGD insult. The *cell surface antigens Ly6A, C* and *E* were down-regulated following LPS/OGD with dobutamine pretreatment. These antigens define very early hematopoietic stem cells. Expression of members of the Ly6 superfamily of genes has also been detected in mouse brain [31]. Expression was found in astrocytes following incubation with cytokines, e.g. *Ifn*-*γ* [30]. To our knowledge these antigens have not previously been implicated in dynamics of inflammatory response within the brain.

Hence, the most remarkeble effect of dobutamine treatment on gene expression in our various experimental paradigms is a strong downregulation of genes associated with inflammation. These genes coded for several chemokines, MHC II antigen, complement protein and several interferon-related genes.

Oxidative stress occurs early following brain ischemia in vivo [32, 33] and can induce tissue damage and neuronal cell death in vitro [34]. Central enzymes in the defense against superoxide anions are the superoxide dismutases (SOD) that convert superoxide anions to hydrogen peroxide, which is further detoxified among others by the thioredoxin-peroxiredoxin system [35]. The protective action of SOD is evident in *Sod*-/- mice that display larger brain infarcts after transient middle cerebral artery occlusion [36]. Indeed, we found that dobutamine pretreatment activates genes involved in the anti-oxidant actions by increasing the expression of anti-oxidants such as Coenzyme Q10. Importantly, the neuronal expression of *thioredoxin reductase* [37], *sulfiredoxin 1* and *sestrin 1* are also up-regulated. The latter genes also mitigate NMDA toxicity [38]. There are three forms of SODs; Cu, Zn SOD (SOD1) which resides in the cytoplasm, MnSOD (SOD2*)* in the mitochondria, and the SOD present in the extracellular matrix (SOD3). An original finding in the present study is the marked up-regulation of *Sod3* after dobutamine treatment in three different experimental paradigms. In addition to the upregulation of genes involved in anti-oxidant actions, dobutamine treatment down-regulates genes, *Vav1, Cybb* and *Ncf1*, associated with the free radical generator NADPH oxidase, which has been implicated in microglial induced acceleration of ischemic neuronal death [39].

Compared to the OGD paradigm, different anti-oxidant genes were regulated in cultures exposed to LPS (Table 1). The combination of dobutamine and LPS showed the upregulation of individual anti-oxidant genes namely thioredoxin domain containing 11, sulfiredoxin 1 homolog, oxidoreductase NAD-binding domain containing 1 and the heat shock response associated genes heat shock 70 kD protein 5 and heat shock protein 1. Upregulation suggests the modulation of the sulfiredoxin/thioredoxin anti-oxidant system, however, further studies will be required to study these cascades and the involvement of dobutamnine more in detail. In addition, the regulation of heat shock response associated genes support previous investigations [30] that dobutamine signalling is involved in the transcriptional control of stress associated heat shock response. Taken together, our data demonstrate the upregulation of genes of the cellular oxidative stress defense system and down-regulation of activators of brain inflammation by dobutamine.

### Regulated genes in two or more experimental paradigms

When comparing gene expression in dobutamine treated slices with the appropriate non-treated groups, essentially all down-regulated genes were related to the inflammatory process. Hence, dobutamine treatment prepares the tissue for a subsequent inflammatory surge by repressing pro-inflammatory mediators. *Ifit2, Iigp1, Rrad, Iigp1*, and *CD72* are genes down-regulated in three experimental conditions by dobutamine. *CD72* is a co-receptor on B-cells that regulates death and survival but has also been found on microglial cells [39]. Interestingly, *SHP*-*1,* a phosphatase regulating the downstream signaling of *CD72* is also down-regulated by dobutamine. The significance of *CD72* is not obvious at present but warrants further investigation.

Genes upregulated by dobutamine encompass antioxidant systems and genes related to survival notably, the antiapoptotic protein Bag-3, and proteins supporting cell survival: inhibin B, Tinf2 and TMBIM1. Notably, two genes were up-regulated in three experimental paradigms, *Slc16a3* [40, 41], a monocarbocylic acid transporter (MCT4) that is selectively expressed in neurons and superoxide dismutase 3. Slc16a3 is a transporter of among others lactate and hence promotes an improved metabolic state in tissue during stress and hypoxia [40]. Of particular interest is the several fold up-regulation of *Sod3*. *Sod3* is bound to the extracellular matrix and prevents the oxidation of hyaluran during inflammation and therefore oxidative damage and migration of inflammatory cells. During degradation of the extracellular matrix during injury and inflammation, *Sod3* is released and the local concentration of *Sod3* decreases and hence the superoxide scavenging capacity of this tissue decreases. It has been shown that *Sod3* is important for the integrity of many tissues, such as lung, kidney pancreas and heart [42–44], and is protective in brain ischemia reperfusion in mouse [45, 46]. *Tnfα* is known to depress the expression of *Sod3* and anti-inflammatory drugs increase levels of *Sod3* [47]. Our data clearly demonstrate show that beta1-adrenoceptor activation provides an alternative route to pharmacologically up-regulate *Sod3* in pathological conditions.

## Conclusions

We conclude that the strong protection provided by dobutamine against LPS/OGD induced tissue damage, is associated with an equally strong downregulation of proinflammatory genes upon LPS challenge and an upregulation of genes associated with the defense against oxidative stress, survival and anabolic anabolic processes. Specifically we propose that *Sod3* has a role in the robust protection. Activation of signaling cascades down stream from the beta1-adrenoceptor may constitute a novel strategy for pharmacologically induced protection of the neonatal brain against hypoxic injury.

## Additional files

**Additional file 1: Table** **S1.** Regulated genes involved in signalling cascades in all experimental groups classified by gene ontology. Abbreviations: D—dobutamine; LPS—lipopolysaccharide; OGD—oxygen–glucose-deprivation.

**Additional file 2: Table** **S2.** Regulated genes involved in transcription in all experimental groups classified by gene ontology. Abbreviations: D—dobutamine; LPS—lipopolysaccharide; OGD—oxygen–glucose-deprivation.

**Additional file 3: Table** **S3.** Regulated genes involved in mechanisms of cell death and cell survival in all experimental groups classified by gene ontology. Abbreviations: D—dobutamine; LPS—lipopolysaccharide; OGD—oxygen–glucose-deprivation.

**Additional file 4: Table** **S4.** Regulated genes involved in metabolism and transport processes in all experimental groups classified by gene ontology. Abbreviations: D—dobutamine; LPS—lipopolysaccharide; OGD—oxygen–glucose-deprivation.

**Additional file 5: Table** **S5.** Regulated genes involved in growth and cell cycle related mechanisms in all experimental groups classified by gene ontology. Abbreviations: D—dobutamine; LPS—lipopolysaccharide; OGD—oxygen–glucose-deprivation.

**Additional file 6: Table** **S6.** Regulated genes involved in development in all experimental groups classified by gene ontology. Abbreviations: D—dobutamine; LPS—lipopolysaccharide; OGD—oxygen–glucose-deprivation.

**Additional file 7: Table** **S7.** Regulated genes involved in cytoskeleton and extracellular matrix dynamics in all experimental groups classified by gene ontology. Abbreviations: D—dobutamine; LPS—lipopolysaccharide; OGD—oxygen–glucose-deprivation.

## References

[1] Grether JK, Nelson KB (1997). Maternal infection and cerebral palsy in infants of normal birth weight. JAMA.

[2] Cai Z, Pang Y, Lin S, Rhodes PG (2003). Differential roles of tumor necrosis factor-alpha and interleukin-1 beta in lipopolysaccharide-induced brain injury in the neonatal rat. Brain Res.

[3] Wang X, Rousset CI, Hagberg H, Mallard C (2006). Lipopolysaccharide-induced inflammation and perinatal brain injury. Semin Fetal Neonatal Med.

[4] Jensen A, Hohmann M, Kunzel W (1987). Dynamic changes in organ blood flow and oxygen consumption during acute asphyxia in fetal sheep. J Dev Physiol.

[5] Blomqvist P, Lindvall O, Wieloch T (1985). Lesions of the locus coeruleus system aggravate ischemic damage in the rat brain. Neurosci Lett.

[6] Gustafson I, Liden A, Wieloch T (1992). Brain cortical tissue levels of noradrenaline and its glycol metabolites: effects of ischemia and postischemic administration of idazoxan. Exp Brain Res.

[7] Mori K, Ozaki E, Zhang B, Yang L, Yokoyama A, Takeda I (2002). Effects of norepinephrine on rat cultured microglial cells that express alpha1, alpha2, beta1 and beta2 adrenergic receptors. Neuropharmacology.

[8] Markus T, Hansson SR, Cronberg T, Cilio C, Wieloch T, Ley D (2010). Beta-Adrenoceptor activation depresses brain inflammation and is neuroprotective in lipopolysaccharide-induced sensitization to oxygen-glucose deprivation in organotypic hippocampal slices. J Neuroinflamm.

[9] Bergles DE, Doze VA, Madison DV, Smith SJ (1996). Excitatory actions of norepinephrine on multiple classes of hippocampal CA1 interneurons. J Neurosci.

[10] Juric DM, Loncar D, Carman-Krzan M (2008). Noradrenergic stimulation of BDNF synthesis in astrocytes: mediation via alpha(1)- and beta(1)/beta(2)-adrenergic receptors. Neurochem Int.

[11] Markus T, Cronberg T, Cilio C, Pronk C, Wieloch T, Ley D (2009). Tumor necrosis factor receptor-1 is essential for LPS-induced sensitization and tolerance to oxygen-glucose deprivation in murine neonatal organotypic hippocampal slices. J Cereb Blood Flow Metab.

[12] Stoppini L, Buchs PA, Muller D (1991). A simple method for organotypic cultures of nervous tissue. J Neurosci Methods.

[13] Cronberg T, Rytter A, Asztely F, Soder A, Wieloch T (2004). Glucose but not lactate in combination with acidosis aggravates ischemic neuronal death in vitro. Stroke.

[14] Jonsson G, Staaf J, Olsson E, Heidenblad M, Vallon-Christersson J, Osoegawa K (2007). High-resolution genomic profiles of breast cancer cell lines assessed by tiling BAC array comparative genomic hybridization. Genes Chromosomes Cancer.

[15] Saal LH, Troein C, Vallon-Christersson J, Gruvberger S, Borg A, Peterson C (2002). BioArray Software Environment (BASE): a platform for comprehensive management and analysis of microarray data. Genome Biol.

[16] Yang YH, Dudoit S, Luu P, Lin DM, Peng V, Ngai J (2002). Normalization for cDNA microarray data: a robust composite method addressing single and multiple slide systematic variation. Nucleic Acids Res.

[17] Markus T, Hansson S, Amer-Wahlin I, Hellstrom-Westas L, Saugstad OD, Ley D (2007). Cerebral inflammatory response after fetal asphyxia and hyperoxic resuscitation in newborn sheep. Pediatr Res.

[18] Ruscher K, Shamloo M, Rickhag M, Ladunga I, Soriano L, Gisselsson L (2011). The sigma-1 receptor enhances brain plasticity and functional recovery after experimental stroke. Brain.

[19] Dirnagl U, Iadecola C, Moskowitz MA (1999). Pathobiology of ischaemic stroke: an integrated view. Trends Neurosci.

[20] Hedtjarn M, Mallard C, Eklind S, Gustafson-Brywe K, Hagberg H (2004). Global gene expression in the immature brain after hypoxia-ischemia. J Cereb Blood Flow Metab.

[21] Rickhag M, Wieloch T, Gido G, Elmer E, Krogh M, Murray J (2006). Comprehensive regional and temporal gene expression profiling of the rat brain during the first 24 h after experimental stroke identifies dynamic ischemia-induced gene expression patterns, and reveals a biphasic activation of genes in surviving tissue. J Neurochem.

[22] Lund S, Christensen KV, Hedtjarn M, Mortensen AL, Hagberg H, Falsig J (2006). The dynamics of the LPS triggered inflammatory response of murine microglia under different culture and in vivo conditions. J Neuroimmunol.

[23] Bjorkbacka H, Fitzgerald KA, Huet F, Li X, Gregory JA, Lee MA (2004). The induction of macrophage gene expression by LPS predominantly utilizes Myd88-independent signaling cascades. Physiol Genomics.

[24] Kawai T, Takeuchi O, Fujita T, Inoue J, Muhlradt PF, Sato S (2001). Lipopolysaccharide stimulates the MyD88-independent pathway and results in activation of IFN-regulatory factor 3 and the expression of a subset of lipopolysaccharide-inducible genes. J Immunol.

[25] Nhu QM, Cuesta N, Vogel SN (2006). Transcriptional regulation of lipopolysaccharide (LPS)-induced Toll-like receptor (TLR) expression in murine macrophages: role of interferon regulatory factors 1 (IRF-1) and 2 (IRF-2). J Endotoxin Res.

[26] Fitzgerald KA, Rowe DC, Barnes BJ, Caffrey DR, Visintin A, Latz E (2003). LPS-TLR4 signaling to IRF-3/7 and NF-kappaB involves the toll adapters TRAM and TRIF. J Exp Med.

[27] Minano FJ, Fernandez-Alonso A, Benamar K, Myers RK, Sancibrian M, Ruiz RM (1996). Macrophage inflammatory protein-1beta (MIP-1beta) produced endogenously in brain during E. coli fever in rats. Eur J Neurosci.

[28] McManus CM, Brosnan CF, Berman JW (1998). Cytokine induction of MIP-1 alpha and MIP-1 beta in human fetal microglia. J Immunol.

[29] Sui Y, Potula R, Dhillon N, Pinson D, Li S, Nath A (2004). Neuronal apoptosis is mediated by CXCL10 overexpression in simian human immunodeficiency virus encephalitis. Am J Pathol.

[30] Roesslein M, Froehlich C, Jans F, Piegeler T, Goebel U, Loop T (2014). Dobutamine mediates cytoprotection by induction of heat shock protein 70 in vitro. Life Sci.

[31] Cray C, Keane RW, Malek TR, Levy RB (1990). Regulation and selective expression of Ly-6A/E, a lymphocyte activation molecule, in the central nervous system. Brain Res Mol Brain Res.

[32] Nelson CW, Wei EP, Povlishock JT, Kontos HA, Moskowitz MA (1992). Oxygen radicals in cerebral ischemia. Am J Physiol.

[33] Peters O, Back T, Lindauer U, Busch C, Megow D, Dreier J (1998). Increased formation of reactive oxygen species after permanent and reversible middle cerebral artery occlusion in the rat. J Cereb Blood Flow Metab.

[34] Bonfoco E, Krainc D, Ankarcrona M, Nicotera P, Lipton SA (1995). Apoptosis and necrosis: two distinct events induced, respectively, by mild and intense insults with *N*-methyl-d-aspartate or nitric oxide/superoxide in cortical cell cultures. Proc Natl Acad Sci USA.

[35] Winyard PG, Moody CJ, Jacob C (2005). Oxidative activation of antioxidant defence. Trends Biochem Sci.

[36] Kondo T, Reaume AG, Huang TT, Carlson E, Murakami K, Chen SF (1997). Reduction of CuZn-superoxide dismutase activity exacerbates neuronal cell injury and edema formation after transient focal cerebral ischemia. J Neurosci.

[37] Bell KF, Hardingham GE (2011). CNS peroxiredoxins and their regulation in health and disease. Antioxid Redox Signal.

[38] Papadia S, Soriano FX, Leveille F, Martel MA, Dakin KA, Hansen HH (2008). Synaptic NMDA receptor activity boosts intrinsic antioxidant defenses. Nat Neurosci.

[39] Toguchi M, Gonzalez D, Furukawa S, Inagaki S (2009). Involvement of Sema4D in the control of microglia activation. Neurochem Int.

[40] Bergersen LH (2007). Is lactate food for neurons? Comparison of monocarboxylate transporter subtypes in brain and muscle. Neuroscience.

[41] Erlichman JS, Hewitt A, Damon TL, Hart M, Kurascz J, Li A (2008). Inhibition of monocarboxylate transporter 2 in the retrotrapezoid nucleus in rats: a test of the astrocyte-neuron lactate-shuttle hypothesis. J Neurosci.

[42] Arcaroli JJ, Hokanson JE, Abraham E, Geraci M, Murphy JR, Bowler RP (2009). Extracellular superoxide dismutase haplotypes are associated with acute lung injury and mortality. Am J Respir Crit Care Med.

[43] Sharma S, Dewald O, Adrogue J, Salazar RL, Razeghi P, Crapo JD (2006). Induction of antioxidant gene expression in a mouse model of ischemic cardiomyopathy is dependent on reactive oxygen species. Free Radic Biol Med.

[44] Folz RJ, Crapo JD (1994). Extracellular superoxide dismutase (SOD3): tissue-specific expression, genomic characterization, and computer-assisted sequence analysis of the human EC SOD gene. Genomics.

[45] Sheng H, Brady TC, Pearlstein RD, Crapo JD, Warner DS (1999). Extracellular superoxide dismutase deficiency worsens outcome from focal cerebral ischemia in the mouse. Neurosci Lett.

[46] Sheng H, Kudo M, Mackensen GB, Pearlstein RD, Crapo JD, Warner DS (2000). Mice overexpressing extracellular superoxide dismutase have increased resistance to global cerebral ischemia. Exp Neurol.

[47] Adachi T, Toishi T, Takashima E, Hara H (2006). Infliximab neutralizes the suppressive effect of TNF-alpha on expression of extracellular-superoxide dismutase in vitro. Biol Pharm Bull.

